# Complete genome sequences of 96 strains of *Ralstonia solanacearum* species complex phylotype I isolated from solanaceous vegetables in Japan

**DOI:** 10.1128/mra.00753-25

**Published:** 2025-10-07

**Authors:** Mitsuo Horita, Ken Naito, Fumio Taguchi-Shiobara, Koji Miyatake, Daisuke Sekine, Kanako Kurita, Yoshimi Shinmura, Kazuhiro Nakaho, Koji Nomiyama, Mamoru Satou

**Affiliations:** 1 Institute for Agro-Environmental Sciences, National Agriculture and Food Research Organization (NARO) 13516, Tsukuba, Ibaraki, Japan; 2 Research Center of Genetic Resources, NARO 13516 , Tsukuba, Ibaraki, Japan; 3 Institute of Crop Science, NARO 13516 , Tsukuba, Ibaraki, Japan; 4 Institute of Vegetable and Floriculture Science, NARO 13516 , Tsu, Mie, Japan; 5 Institute for Plant Protection, NARO 13516 , Tsukuba, Ibaraki, Japan; Rochester Institute of Technology, Rochester, New York, USA

**Keywords:** bacterial wilt, *Ralstonia solanacearum *species complex, solanaceous vegetables

## Abstract

Bacterial wilt disease, caused by *Ralstonia solanacearum* species complex phylotype I, severely affects the cultivation of solanaceous vegetables (tomato, eggplant, etc.) in Japan. In this study, we report the complete genome sequences of 96 strains of the pathogen isolated from solanaceous vegetables in various parts of Japan.

## ANNOUNCEMENT

Bacterial wilt disease, caused by *Ralstonia solanacearum* species complex (RSSC) (1, 2), is a major bacterial disease worldwide (3). In Japan, RSSC belonging to phylotype I causes severe damage to solanaceous vegetables (4). Here, we determined the complete genome sequences of 96 strains of RSSC phylotype I isolated from diseased solanaceous vegetables (tomato, eggplant, peppers, and potatoes) from 32 prefectures of Japan (Table 1).

**TABLE 1:** List of RSSC phylotype I strain^*a*^

No.	MAFF no.r^*b*^	Host, location, year	BioSample	DRA accession no. (short read) (long read)	No. of reads	*N*_50_ value (bp)	Genomic DNA^*c*^	Accession no.	Genome size (bp)	Coverage (×)	CDS^*d*^
1	106244	Eggplant, Nara, 1981	SAMD00889216	DRR657716	1,335,612						
				DRR657717	28,068	22,651	Total		5,877,558	85	5,159
							C	AP041399	3,859,863		3,569
							M	AP041400	2,017,695		1,590
2	106251	Eggplant, Osaka, 1981	SAMD01229170	DRR667423	1,553,992						
				DRR667424	16,173	31,826	Total		5,877,554	80	5,161
							C	AP041401	3,859,856		3,571
							M	AP041402	2,017,698		1,590
3	724001	Potato, Nara, 1982	SAMD01229171	DRR667425	1,483,493						
				DRR667426	16,028	34,574	Total		5,951,892	79	5,251
							C	AP041403	3,975,213		3,695
							M	AP041404	1,976,679		1,556
4	724002	Eggplant, Tottori, 1982	SAMD01229172	DRR667427	1,781,508						
				DRR667428	28,389	27,233	Total		5,787,814	101	5,052
							C	AP041405	3,825,018		3,530
							M	AP041406	1,962,796		1,522
5	724003	Eggplant, Okayama, 1982	SAMD01229173	DRR667429	1,905,699						
				DRR667430	20,034	29,362	Total		5,787,820	92	5,055
							C	AP041407	3,825,025		3,533
							M	AP041408	1,962,795		1,522
6	106252	Eggplant, Fukuoka, 1982	SAMD01229174	DRR667431	2,276,757						
				DRR667432	14,847	30,730	Total		5,784,412	88	5,060
							C	AP041409	3,817,949		3,516
							M	AP041410	1,966,463		1,544
7	724004	Tomato, Hyogo, 1982	SAMD01229175	DRR667433	1,678,190						
				DRR667434	13,511	34,538	Total		5,924,327	77	5,206
							C	AP041411	3,930,316		3,641
							M	AP041412	1,994,011		1,565
8	106253	Eggplant, Hiroshima, 1982	SAMD01229176	DRR667435	1,732,297						
				DRR667436	14,323	26,925	Total		5,854,653	71	5,166
							C	AP041413	3,873,661		3,618
							M	AP041414	1,980,992		1,548
9	724005	Eggplant, Osaka, 1982	SAMD01229177	DRR667437	1,950,644						
				DRR667438	28,234	24,903	Total		5,860,690	106	5,145
							C	AP041415	3,859,476		3,565
							M	AP041416	2,001,214		1,580
10	724008	Tomato, Shizuoka, 1982	SAMD01229178	DRR667439	1,431,216						
				DRR667440	14,795	28,872	Total		5,751,182	67	5,000
							C	AP041417	3,748,644		3,431
							M	AP041418	2,002,538		1,569
11	106245	Tomato, Mie, 1982	SAMD01229179	DRR667441	1,530,502						
				DRR667442	27,622	30,653	Total		5,813,616	102	5,083
							C	AP041419	3,750,393		3,452
							M	AP041420	2,063,223		1,631
12	724009	Tomato, Ibaraki, 1982	SAMD01229180	DRR667443	1,689,531						
				DRR667444	17,656	26,052	Total		5,949,963	77	5,244
							C	AP041421	3,889,075		3,599
							M	AP041422	2,060,888		1,645
13	724010	Eggplant, Gunma, 1982	SAMD01229181	DRR667445	1,614,600						
				DRR667446	12,915	37,791	Total		5,830,308	78	5,100
							C	AP041423	3,837,594		3,541
							M	AP041424	1,992,714		1,559
14	311869	Tomato, Kochi, 1982	SAMD01229182	DRR667447	1,833,824						
				DRR667448	15,500	38,761	Total		5,826,993	92	5,111
							C	AP041425	3,864,193		3,588
							M	AP041426	1,962,800		1,523
15	311870	Tomato, Kochi, 1982	SAMD01229183	DRR667449	1,755,668						
				DRR667450	16,049	35,638	Total		5,864,197	91	5,164
							C	AP041427	3,877,464		3,602
							M	AP041428	1,986,733		1,562
16	311871	Eggplant, Kochi, 1982	SAMD01229184	DRR667451	1,850,141						
				DRR667452	29,282	28,965	Total		5,829,152	113	5,111
							C	AP041429	3,864,274		3,588
							M	AP041430	1,964,878		1,523
17	311872	Tomato, Kagoshima, 1982	SAMD01229185	DRR667453	1,866,691						
				DRR667454	12,733	34,620	Total		5,797,396	81	5,069
							C	AP041431	3,834,609		3,545
							M	AP041432	1,962,787		1,524
18	311873	Eggplant, Saga, 1982	SAMD01229186	DRR667455	1,733,634						
				DRR667456	16,646	29,176	Total		5,864,206	82	5,129
							C	AP041433	3,901,396		3,604
							M	AP041434	1,962,810		1,525
19	311874	Tomato, Osaka, 1982	SAMD01229187	DRR667457	1,762,344						
				DRR667458	25,586	31,318	Total		5,957,112	108	5,265
							C	AP041435	3,981,626		3,701
							M	AP041436	1,975,486		1,564
20	311875	Tomato, Shimane, 1982	SAMD01229188	DRR667459	1,981,995						
				DRR667460	25,783	28,512	Total		5,970,545	107	5,229
							C	AP041437	3,975,104		3,669
							M	AP041438	1,995,441		1,560
21	106254	Eggplant, Wakayama, 1982	SAMD01229189	DRR667461	1,885,022						
				DRR667462	33,480	22,615	Total		5,859,969	111	5,144
							C	AP041439	3,866,563		3,583
							M	AP041440	1,993,406		1,561
22	311876	Eggplant, Tokyo, 1982	SAMD01229190	DRR667463	2,270,174						
				DRR667464	10,352	43,728	Total		5,733,050	89	4,994
							C	AP041441	3,746,404		3,434
							M	AP041442	1,986,646		1,560
23	311877	Tomato, Gunma, 1982	SAMD01229191	DRR667465	1,988,279						
				DRR667466	13,138	38,407	Total		6,072,814	85	5,342
							C	AP041443	4,001,663		3,716
							M	AP041444	2,071,151		1,626
24	311878	Eggplant, Saitama, 1982	SAMD01229192	DRR667467	1,947,749						
				DRR667468	12,913	41,801					
							C	AP041445	5,824,784	90	5,099
25	311879	Tomato, Nagano, 1982	SAMD01229193	DRR667469	1,538,210						
				DRR667470	13,940	33,140	Total		5,992,854	75	5,242
							C	AP041446	3,920,407		3,614
							M	AP041447	2,072,447		1,628
26	311880	Tomato, Yamaguchi, 1982	SAMD01229194	DRR667471	1,460,559						
				DRR667472	27,072	27,382	Total		5,909,876	95	5,176
							C	AP041448	3,916,609		3,619
							M	AP041449	1,993,267		1,557
27	311881	Tomato, Yamaguchi, 1982	SAMD01229195	DRR667473	731,214						
				DRR667474	15,525	33,813	Total		5,870,348	58	5,165
							C	AP041450	3,867,810		3,567
							M	AP041451	2,002,538		1,598
28	311882	Tomato, Yamaguchi, 1982	SAMD01229196	DRR667475	772,604						
				DRR667476	22,148	28,922	Total		6,008,490	67	5,259
							C	AP041452	3,933,133		3,626
							M	AP041453	2,075,357		1,633
29	311883	Tomato, Kanagawa, 1982	SAMD01229197	DRR667477	1,713,409						
				DRR667478	17,798	28,791	Total		5,878,557	81	5,207
							C	AP041454	3,906,617		3,638
							M	AP041455	1,971,940		1,569
30	311884	Eggplant, Yamanashi, 1982	SAMD01229198	DRR667479	1,537,854						
				DRR667480	34,306	26,025	Total		5,931,772	109	5,243
							C	AP041456	3,950,773		3,691
							M	AP041457	1,980,999		1,552
31	311885	Tomato, Oita, 1982	SAMD01229199	DRR667481	1,826,051						
				DRR667482	34,214	21,775	Total		6,013,698	108	5,292
							C	AP041458	3,938,464		3,653
							M	AP041459	2,075,234		1,639
32	311886	Tomato, Oita, 1982	SAMD01229200	DRR667483	1,588,115						
				DRR667484	19,490	31,633	Total		5,786,151	87	5,048
							C	AP041460	3,804,279		3,491
							M	AP041461	1,981,872		1,557
33	311887	Pepper, Oita, 1982	SAMD01229201	DRR667485	1,736,489						
				DRR667486	22,251	34,896	Total		5,778,998	106	5,046
							C	AP041462	3,816,377		3,505
							M	AP041463	1,962,621		1,541
34	311888	Tomato, Ehime, 1982	SAMD01229202	DRR667487	1,854,888						
				DRR667488	13,040	32,986	Total		5,762,479	79	5,009
							C	AP041464	3,773,364		3,452
							M	AP041465	1,989,115		1,557
35	106255	Tomato, Kumamoto, 1982	SAMD01229203	DRR667489	1,937,994						
				DRR667490	19,427	34,883	Total		5,794,303	99	5,067
							C	AP041466	3,827,815		3,524
							M	AP041467	1,966,488		1,543
36	311889	Eggplant, Fukuoka, 1982	SAMD01229204	DRR667491	1,882,112						
				DRR667492	23,983	31,598	Total		5,962,297	107	5,232
							C	AP041468	3,938,465		3,648
							M	AP041469	2,023,832		1,584
37	311890	Eggplant, Shizuoka, 1982	SAMD01229205	DRR667493	1,401,814						
				DRR667494	18,766	30,727	Total		6,063,933	79	5,359
							C	AP041470	4,004,902		3,729
							M	AP041471	2,059,031		1,630
38	311891	Tomato, Aichi, 1982	SAMD01229206	DRR667495	1,650,554						
				DRR667496	13,791	28,985	Total		5,923,037	72	5,198
							C	AP041472	3,955,427		3,663
							M	AP041473	1,967,610		1,535
39	311892	Potato, Nagasaki, 1982	SAMD01229207	DRR667497	1,801,689						
				DRR667498	22,324	36,076	Total		5,772,947	105	5,027
							C	AP041474	3,783,865		3,465
							M	AP041475	1,989,082		1,562
40	311893	Tomato, Kanagawa, 1982	SAMD01229208	DRR667499	2,165,272						
				DRR667500	18,787	32,290	Total		5,878,666	103	5,198
							C	AP041476	3,906,727		3,635
							M	AP041477	1,971,939		1,563
41	311894	Tomato, Shizuoka, 1982	SAMD01229209	DRR667501	1,197,862						
				DRR667502	11,844	36,345	Total		5,968,701	64	5,275
							C	AP041478	3,994,945		3,738
							M	AP041479	1,973,756		1,537
42	311895	Eggplant, Saga, 1983	SAMD01229210	DRR667503	1,341,336						
				DRR667504	11,650	38,383	Total		5,746,959	66	5,025
							C	AP041480	3,791,116		3,501
							M	AP041481	1,955,843		1,524
43	311896	Tomato, Shizuoka, 1983	SAMD01229211	DRR667505	1,966,548						
				DRR667506	21,509	28,928	Total		5,863,271	96	5,125
							C	AP041482	3,862,164		3,553
							M	AP041483	2,001,107		1,572
44	311897	Tomato, Ehime, 1983	SAMD01229212	DRR667507	1,155,976						
				DRR667508	13,547	36,611	Total		5,968,507	66	5,249
							C	AP041484	3,981,713		3,684
							M	AP041485	1,986,794		1,565
45	311898	Eggplant, Kyoto, 1983	SAMD01229213	DRR667509	1,422,502						
				DRR667510	13,481	40,538	Total		5,896,643	77	5,172
							C	AP041486	3,904,732		3,609
							M	AP041487	1,991,911		1,563
46	311899	Tomato, Fukui, 1983	SAMD01229214	DRR667511	1,999,562						
				DRR667512	22,029	38,589	Total		5,737,741	113	4,989
							C	AP041488	3,737,704		3,411
							M	AP041489	2,000,037		1,578
47	106256	Eggplant, Oita, 1983	SAMD01229215	DRR667513	1,309,706						
				DRR667514	12,824	38,940	Total		5,816,819	68	5,084
							C	AP041490	3,818,903		3,512
							M	AP041491	1,997,916		1,572
48	311900	Eggplant, Kyoto, 1983	SAMD01229216	DRR667515	1,672,113						
				DRR667516	14,878	26,998	Total		5,788,130	70	5,057
							C	AP041492	3,824,157		3,533
							M	AP041493	1,963,973		1,524
49	311901	Pepper, Kochi, 1983	SAMD01229217	DRR667517	1,590,209						
				DRR667518	14,606	32,814	Total		6,167,988	76	5,431
							C	AP041494	4,104,406		3,811
							M	AP041495	2,063,582		1,620
50	106257	Tomato, Kochi, 1983	SAMD01229218	DRR667519	1,453,794						
				DRR667520	23,817	33,865	Total		5,828,644	96	5,137
							C	AP041496	3,713,932		3,461
							M	AP041497	2,114,712		1,676
51	311902	Tomato, Kochi, 1983	SAMD01229219	DRR667521	1,562,679						
				DRR667522	11,731	33,581	Total		5,707,225	69	5,003
							C	AP041498	3,674,427		3,407
							M	AP041499	2,032,798		1,596
52	106204	Eggplant, Kochi, 1983	SAMD01229220	DRR667523	1,725,633						
				DRR667524	27,196	30,776	Total		5,827,089	108	5,110
							C	AP041500	3,864,280		3,588
							M	AP041501	1,962,809		1,522
53	106205	Eggplant, Kochi, 1983	SAMD01229221	DRR667525	1,450,744						
				DRR667526	26,518	29,537	Total		5,825,193	94	5,112
							C	AP041502	3,864,277		3,587
							M	AP041503	1,960,916		1,525
54	106258	Eggplant, Kochi, 1983	SAMD01229222	DRR667527	1,566,992						
				DRR667528	11,251	34,241	Total		5,763,068	68	5,035
							C	AP041504	3,825,313		3,532
							M	AP041505	1,937,755		1,503
55	106206	Eggplant, Fukuoka, 1984	SAMD01229223	DRR667529	1,522,992						
				DRR667530	33,363	28,549	Total		5,850,262	88	5,128
							C	AP041506	3,814,727		3,534
							M	AP041507	2,035,535		1,594
56	106207	Tomato, Hiroshima, 1984	SAMD01229224	DRR667531	1,226,118						
				DRR667532	21,582	39,106	Total		5,762,977	91	5,033
							C	AP041508	3,826,397		3,531
							M	AP041509	1,936,580		1,502
57	106208	Eggplant, Hiroshima, 1984	SAMD01229225	DRR667533	1,987,491						
				DRR667534	20,421	26,959	Total		5,771,297	92	5,016
							C	AP041510	3,781,035		3,458
							M	AP041511	1,990,262		1,558
58	106209	Tomato, Hiroshima, 1984	SAMD01229226	DRR667535	1,451,726						
				DRR667536	19,721	27,117	Total		5,959,154	78	5,265
							C	AP041512	3,984,380		3,707
							M	AP041513	1,974,774		1,558
59	106210	Eggplant, Nara, 1984	SAMD01229227	DRR667537	1,709,384						
				DRR667538	13,915	35,260	Total		6,073,546	80	5,294
							C	AP041514	4,012,282		3,684
							M	AP041515	2,061,264		1,610
60	106211	Eggplant, Kyoto, 1984	SAMD01229228	DRR667539	1,776,768						
				DRR667540	25,765	28,296	Total		5,788,129	102	5,053
							C	AP041516	3,824,159		3,528
							M	AP041517	1,963,970		1,525
61	106212	Tomato, Kyoto, 1984	SAMD01229229	DRR667541	1,778,239						
				DRR667542	14,365	29,061	Total		5,763,643	77	5,020
							C	AP041518	3,774,554		3,461
							M	AP041519	1,989,089		1,559
62	106213	Eggplant, Hyogo, 1985	SAMD01229230	DRR667543	2,114,408						
				DRR667544	16,417	35,716	Total		5,887,165	94	5,208
							C	AP041520	3,890,103		3,637
							M	AP041521	1,997,062		1,571
63	106214	Tomato, Tottori, 1985	SAMD01229231	DRR667545	1,937,572						
				DRR667546	35,325	29,186	Total		5,947,209	123	5,214
							C	AP041522	3,956,462		3,655
							M	AP041523	1,990,747		1,559
64	106215	Tomato, Nara, 1985	SAMD01229232	DRR667547	1,688,859						
				DRR667548	22,723	29,562	Total		5,972,019	95	5,237
							C	AP041524	3,977,633		3,675
							M	AP041525	1,994,386		1,562
65	106216	Eggplant, Kyoto, 1985	SAMD01229233	DRR667549	2,064,695						
				DRR667550	22,678	30,062	Total		5,991,520	105	5,289
							C	AP041526	3,975,237		3,700
							M	AP041527	2,016,283		1,589
66	106217	Eggplant, Hiroshima, 1987	SAMD01229234	DRR667551	1,702,280						
				DRR667552	20,557	26,192	Total		5,682,684	83	4,945
							C	AP041528	3,718,867		3,404
							M	AP041529	1,963,817		1,541
67	106218	Eggplant, Mie, 2020	SAMD01229235	DRR667553	1,536,690						
				DRR667554	31,179	30,994					
							C	AP041530	5,872,279	113	5,134
68	311908	Pepper, Kyoto, 2007	SAMD01229236	DRR667555	1,838,524						
				DRR667556	14,702	31,244	Total		5,983,525	80	5,242
							C	AP041531	3,913,610		3,615
							M	AP041532	2,069,915		1,627
69	311931	Pepper, Kyoto, 1995	SAMD01229237	DRR667557	1,858,227						
				DRR667558	19,055	30,168	Total		5,951,512	90	5,241
							C	AP041533	3,955,129		3,666
							M	AP041534	1,996,383		1,575
70	311910	Pepper, Kochi, 1997	SAMD01229238	DRR667559	1,425,276						
				DRR667560	42,147	23,109	Total		6,157,846	113	5,431
							C	AP041535	4,092,890		3,807
							M	AP041536	2,064,956		1,624
71	311909	Pepper, Kochi, 1997	SAMD01229239	DRR667561	1,994,216						
				DRR667562	21,581	40,481	Total		6,171,365	116	5,431
							C	AP041537	4,088,548		3,800
							M	AP041538	2,082,817		1,631
72	211266	Eggplant, Hiroshima, 1973	SAMD01229240	DRR667563	2,082,029						
				DRR667564	23,416	38,033	Total		5,861,269	116	5,155
							C	AP041539	3,852,230		3,577
							M	AP041540	2,009,039		1,578
73	211282	Eggplant, Kochi, 1993	SAMD01229241	DRR667565	1,584,583						
				DRR667566	11,578	32,294	Total		5,827,957	69	5,109
							C	AP041541	3,865,143		3,586
							M	AP041542	1,962,814		1,523
74	331041	Tomato, Tochigi, 2011	SAMD01229242	DRR667567	1,892,787						
				DRR667568	15,629	29,300	Total		5,801,374	83	5,102
							C	AP041543	3,878,807		3,586
							M	AP041544	1,922,567		1,516
75	331042	Eggplant, Tochigi, 2011	SAMD01229243	DRR667569	1,605,001						
				DRR667570	18,229	35,134	Total		5,872,477	88	5,149
							C	AP041545	3,891,892		3,593
							M	AP041546	1,980,585		1,556
76	331046	Tomato, Tochigi, 2011	SAMD01229244	DRR667571	1,740,081						
				DRR667572	15,995	30,694	Total		5,901,312	81	5,163
							C	AP041547	3,990,785		3,675
							M	AP041548	1,910,527		1,488
77	311906	Eggplant, Osaka, 1997	SAMD01229245	DRR667573	1,947,361						
				DRR667574	48,143	27,757	Total		5,796,534	152	5,101
							C	AP041549	3,821,023		3,547
							M	AP041550	1,975,511		1,554
78	311907	Eggplant, Osaka, 1997	SAMD01229246	DRR667575	1,748,226						
				DRR667576	15,535	38,185	Total		5,795,064	84	5,108
							C	AP041551	3,818,341		3,550
							M	AP041552	1,976,723		1,558
79	311855	Eggplant, Okayama, 2017	SAMD01229247	DRR667577	2,092,244						
				DRR667578	19,426	30,903	Total		5,793,865	98	5,055
							C	AP041553	3,800,875		3,496
							M	AP041554	1,992,990		1,559
80	107632	Tomato, Nara, 1981	SAMD01229248	DRR667579	1,692,505						
				DRR667580	17,549	30,948	Total		5,911,011	86	5,192
							C	AP041555	3,914,014		3,624
							M	AP041556	1,996,997		1,568
81	106259	Eggplant, Osaka, 1981	SAMD01229249	DRR667581	1,780,511						
				DRR667582	17,391	29,151	Total		5,873,679	83	5,148
							C	AP041557	3,879,681		3,587
							M	AP041558	1,993,998		1,561
82	106260	Tomato, Nara, 1982	SAMD01229250	DRR667583	1,760,106						
				DRR667584	24,795	28,270	Total		5,951,923	98	5,256
							C	AP041559	3,975,217		3,696
							M	AP041560	1,976,706		1,560
83	106261	Tomato, Gunma, 1982	SAMD01229251	DRR667585	1,810,225						
				DRR667586	30,819	26,464	Total		5,952,484	110	5,242
							C	AP041561	3,892,397		3,603
							M	AP041562	2,060,087		1,639
84	211268	Eggplant, Kochi, 1982	SAMD01229252	DRR667587	1,810,876						
				DRR667588	19,156	28,445	Total		6,103,685	88	5,384
							C	AP041563	4,038,488		3,759
							M	AP041564	2,065,197		1,625
85	106263	Eggplant, Kochi, 1982	SAMD01229253	DRR667589	1,625,426						
				DRR667590	19,853	34,224	Total		5,995,488	92	5,258
							C	AP041565	3,918,289		3,621
							M	AP041566	2,077,199		1,637
86	211267	Tomato, Shimane, 1982	SAMD01229254	DRR667591	1,642,696						
				DRR667592	19,728	27,290	Total		5,882,274	83	5,157
							C	AP041567	3,894,796		3,598
							M	AP041568	1,987,478		1,559
87	106264	Tomato, Hyogo, 1982	SAMD01229255	DRR667593	2,056,662						
				DRR667594	25,219	26,804	Total		5,986,207	105	5,296
							C	AP041569	3,853,259		3,539
							M	AP041570	2,132,948		1,757
88	106265	Tomato, Nagano, 1982	SAMD01229256	DRR667595	1,924,721						
				DRR667596	19,146	29,145					
							C	AP041571	5,824,791	93	5,088
89	106266	Tomato, Shizuoka, 1982	SAMD01229257	DRR667597	1,892,494						
				DRR667598	14,684	33,562	Total		5,931,771	85	5,241
							C	AP041572	3,950,773		3,693
							M	AP041573	1,980,998		1,548
90	106267	Eggplant, Fukuoka, 1983	SAMD01229258	DRR667599	1,911,990						
				DRR667600	25,670	25,379	Total		5,791,512	101	5,066
							C	AP041574	3,826,834		3,526
							M	AP041575	1,964,678		1,540
91	106268	Eggplant, Kochi, 1983	SAMD01229259	DRR667601	1,534,680						
				DRR667602	14,826	37,526	Total		6,087,572	83	5,370
							C	AP041576	4,022,743		3,743
							M	AP041577	2,064,829		1,627
92	106269	Tomato, Shiga, 1984	SAMD01229260	DRR667603	1,771,150						
				DRR667604	22,614	29,405	Total		5,935,647	97	5,249
							C	AP041578	3,969,107		3,698
							M	AP041579	1,966,540		1,551
93	211269	Eggplant, Nara, 1985	SAMD01229261	DRR667605	2,042,781						
				DRR667606	14,316	34,211	Total		5,872,513	90	5,157
							C	AP041580	3,853,604		3,566
							M	AP041581	2,018,909		1,591
94	311771	Tomato, Kumamoto, 2017	SAMD01229262	DRR667607	1,044,996						
				DRR667608	23,339	22,171	Total		5,788,974	69	5,056
							C	AP041582	3,826,179		3,534
							M	AP041583	1,962,795		1,522
95	311772	Tomato, Saitama, 2017	SAMD01229263	DRR667609	1,089,342						
				DRR667610	22,870	24,187	Total		5,833,954	73	5,114
							C	AP041584	3,784,234		3,504
							M	AP041585	2,049,720		1,610
96	311800	Tomato, Shizuoka, 2018	SAMD01229264	DRR667611	1,180,068						
				DRR667612	15,130	26,016					
							C	AP041586	5,962,481	62	5,257

^*a*^ Based on previous reports (1, 2, 4).

^*b*^ Genebank, Research Center of Genetic Resources, NARO (Tsukuba, Japan).

^*c*^ C, chromosome; M, megaplasmid.

^*d*^ CDS, coding DNA sequences.

These strains were preserved as freeze-dried cells at the Genebank of the National Agriculture and Food Research Organization (NARO, Tsukuba, Japan). A single colony isolated on triphenyl tetrazolium chloride medium (5) was cultured in casamino acid-peptone-glucose broth (5) overnight at 30°C. Genomic DNA was extracted using NucleoBond HMW DNA (Macherey-Nagel, Düren, Germany) according to the manufacturer’s instructions. The extracted DNA was subjected to short- and long-read sequencing. For short reads, we generated paired-end libraries (fragment size, ~550 bp) using the Collibri ES DNA Library Prep Kit for Illumina Systems (ThermoFisher Scientific, Waltham, MA). For adaptors, the KAPA HyperPrep Kit with KAPA Universal Adapter and KAPA UDI Primer Mixes_1-96 (Nippon Genetics, Tokyo, Japan) was used. The pooled libraries were sequenced on an Illumina NovaSeq 6000 System (Illumina, San Diego, CA), generating 150-bp paired-end reads. Adapter sequences, low-quality reads (reads in which the number of bases with Qphred ≤ 5 accounted for more than 50% of the total reads), and >10% reads with nucleotide sequence unknown were removed by Fastp (ver. 0.23.4) (6). For long reads, genomic DNA was processed using a Rapid Barcoding Kit (SQK-RBK109-96; Oxford Nanopore Technologies, Oxford, UK). The pooled libraries were sequenced using PromethION 24 (Oxford Nanopore Technologies) with R9.4.1 flow cells under a high-accuracy mode of MinKNOW-4.3.1. Quality scores were measured using a logarithmic Phred scale. Reads shorter than 1 kbp or below the mean quality score of 7 were removed by SeqKit2 (ver. 2.8.2) (7) as failed reads. Library preparation and sequencing were performed at the Research Center of Genetic Resources, NARO (Tsukuba, Japan). Hybrid assembly with long and short reads was performed using Flye (ver. 2.9) (8), NECAT (ver. 0.0.1) (9), and Raven (ver. 1.7.0) (10) and phased into a consensus using Trycycler (ver. 0.5.2) (11). Trycyler searched for *dnaA* alleles within each completed replicon and circularized the sequences. The phased assemblies were further polished using medaka-1.7.2 (https://github.com/nanoporetech/medaka) with long reads and hypo-1.0.3 (12) with short reads. Genome annotation was performed using the DFAST program (ver. 1.6.0) (13). Unless otherwise stated, default parameters were used, but the expected genome size was set to 6 Mbp.

The characteristics of the sequenced genomes are shown in Table 1. The GC content of the test strains ranged from 66.7% to 67.0%. These results are similar to those previously reported for RSSC strains (3, 14).

## Data Availability

The complete sequences of the 96 RSSC strains have been deposited in the DNA Data Bank of Japan (DDBJ) under BioProject accession number PRJDB20372. The BioSample accession numbers, DRA accession numbers (short- and long-read archives), and genome nucleotide accession numbers of each strain are shown in Table 1.
